# Oxytranscriptome of soybean seedlings under short-term cadmium treatment

**DOI:** 10.1038/s41598-025-09324-0

**Published:** 2025-07-13

**Authors:** Jarosław Gzyl, Marek Żywicki, Joanna Deckert, Mikołaj Lichocki, Yara Skafe, Jacek Bąk, Wojciech Roszka, Yang Shen, Xiaoli Sun, Jagna Chmielowska-Bąk

**Affiliations:** 1 Department of Plant Ecophysiology, Institute of Experimental Biology, Faculty of Biology, Adam Mickiewicz University, Poznań, Poland; 2 Department of Computational Biology, Institute of Molecular Biology, Faculty of Biology, Adam Mickiewicz University, Poznań, Poland; 3 Afyon Kocatepe University, Afyonkarahisar, Turkey; 4 Collegium Da Vinci, Poznań, Poland; 5 Department of Statistic, Institute of Informatics and Quantitative Economics, Poznań University of Economics and Business, Poznań, Poland; 6 Crop Stress Molecular Biology Laboratory, Heilongjiang Bayi Agricultural University, Daqing, 163319 Heilongjiang China

**Keywords:** RNA oxidation, 8-OHG, 8-hydroxyguanosine, Epitranscriptomics, Metals, Cadmium, Reactive oxygen species, Plant molecular biology, Plant stress responses, Abiotic, Plant sciences

## Abstract

Despite the important role of reactive oxygen species (ROS) in plant stress response, little is known about their effects on RNA. Present study focuses on RNA oxidation in soybean seedlings exposed for a short time to cadmium (1–3 h, 10 mg/L Cd). Treatment with the metal had no effect on the level of common oxidative stress markers: superoxide anion accumulation, lipid peroxidation, protein carbonylation and reduced/oxidized glutathione ratio. On the other hand, 2 h-long exposure to Cd induced the formation of the most frequent oxidative modification of RNA, 8-hydroxyguanosine (8-OHG). The immunohistochemical detection of 8-OHG in the root cross sections demonstrated that this modification is accumulated in the inner cortex tissue, endodermis, protoxylem and phloem, and co-localizes with ROS. The sequencing of 8-OHG enriched poly(A)RNA revealed that transcripts differ in their susceptibility to the oxidation. The highly oxidized transcripts (HOTs) are associated with signalling, stress response and proteins folding. In turn, the lowly oxidized transcripts (LOTs) are involved in mitochondria functioning, response to stimuli and gibberellic acid (GA) metabolism.

**Supplementary Information:**

The online version contains supplementary material available at 10.1038/s41598-025-09324-0.

## Introduction

Reactive oxygen species (ROS) are highly reactive forms of oxygen, which include e.g. hydrogen peroxide (H_2_O_2_), superoxide anion (O_2_^−·^), singlet oxygen (^1^O_2_) and hydroxyl radical (·OH). They differ in the sites of production, reactivity and lifetime and, in consequence, also in their functions in plants. Presently, ROS, in particularly H_2_O_2_, are perceived as important signalling molecules engaged in the developmental processes, retrograde signalling and stress response^1,2^. However, ROS excess might also lead to oxidative stress and negatively impact plant functioning^3^. Moreover, ROS-dependent oxidation of biomolecules might have dual effects. On the one hand, it can lead to the damage of biomolecules and associated impaired functioning. On the other hand, the products of protein, lipid and RNA oxidation might be involved in signalling events^4^.

Ribonucleic acids are fundamental in the regulation of genes expression. Their metabolism is modulated through chemical modifications such as methylation of adenosine at N6 position (m6A), methylation of cytosine (5mC) or oxidation and nitration of guanosine (8-OHG and 8-NO_2_-G, respectively). These modifications, in particular m6A and 5mC, were shown to regulate transcripts stability, splicing, alternative polyadenylation, translocation, interactions with other molecules and translation efficiency, greatly affecting the functioning of organisms [reviewed in^5–7^]. The most frequent ROS-dependent oxidative modification of RNA is the formation of 8-hydroxyguanosine (8-OHG). Despite the fact that ROS are important in plants stress response, the 8-OHG formation, metabolism and roles in plants under optimal and unfavourable conditions are poorly examined.

Initial studies on 8-OHG were focused on neurodegenerative disease in animal models. The results revealed a significant increase in 8-OHG levels in RNA isolated from postmortem brains of patients suffering from Alzheimer’s disease^8^. In subsequent studies, 8-OHG accumulation was noted also in other disorders including Parkinson’s disease, amyotrophic lateral sclerosis (ALS), diabetes and cancers^9^. Moreover, the process of aging is associated with an increase in urinary 8-OHG levels^10^. More detailed insights into the role of this modification in pathogenesis showed that 8-OHG formation precedes the development of disease symptoms, is selective, limited to certain sets of transcripts and leads to translational errors^11–13^. The 8-OHG effects on translation were studied more in detail in the in vitro model. It was evidenced that the presents of 8-OHG in mRNA leads to hampered translation independently of the position in the codon. This effect results from alerted interactions between 8-OHG enriched mRNA and the tRNA molecules^14^.

In the case of plants, the first published studies on 8-OHG concerned its involvement in seeds transition from dormant to non-dormant state^15,16^. Similar as in the case of animals, also in seeds 8-OHG occurred in specific sets of transcripts confirming the selectiveness of RNA oxidation. In addition, it has been shown that 8-OHG formation leads to decrease in the level of encoded proteins. In wheat seeds, some of the oxidized transcripts encoded inhibitors of the storage material mobilization. Thus, in this case, oxidation of specific transcripts may lead to decrease in the level of inhibitor proteins and in consequence facilitate mobilization of seeds storage material – a crucial process during seeds germination^15,16^. Our previous studies showed for the first time stress dependent oxidation of RNA in plants. Accumulation of 8-OHG in total and poly(A)RNA was noted in response to the short-term Cd treatment^17^. These findings were supported by further reports indicating 8-OHG accumulation in response to other metals (copper and lead)^18^, and aphid^19^ and nematode infection^20^.

Despite the increasing number of reports on 8-OHG formation in plants, little is known about its biological effects. The aim of this study is getting deeper insights into the effects of Cd-dependent RNA 8-OHG formation in the roots of soybean seedlings. Previous report showed that 8-OHG accumulation occurred after 3 h of treatment with Cd at the concentration of 10 mg/L^17^. Thus, the study is focused on the early metal response – the first three hours of exposure to Cd. The study includes quantification of 8-OHG level in RNA in relation to the general oxidative status, immunohistochemical detection of 8-OHG in plant tissues and identification of 8-OHG enriched transcripts by the means of immunoprecipitation and RNA sequencing.

## Methods

### Cultivation and treatment procedures

Soybean (*Glycine max* L. cv. Nawiko) seeds were obtained from the Department of Genetics and Plant Breeding of the University of Life Sciences in Poznań, Poland. The seeds were surface sterilized (5 min in 75% ethanol and 10 min in 1% sodium hypochlorite), washed under running water for 30 min and imbibed for 3–4 h in tap water. The seeds were germinated for 3 days in growth chamber (21–22 °C in the dark) on trays lined with two layers of lignin and one layer of filter paper moistened with water and covered with aluminum foil. Thereafter, seedlings selected on the basis of similar root length were transferred to glass Petri dishes (10 cm diameter) with roots placed between two layers of filter paper in cutout holes. Seedlings were then treated with 5 mL of distilled water (control) or CdCl_2_ with Cd at the concentration of 10 mg/L (corresponding to 89 µM, pH = 7.3). The metal concentration was chosen on the basis of previous study^17^. After 2 h of treatment the roots were cut off on ice, snap frozen in liquid nitrogen and stored at − 80 °C.

### Quantification of the Cd level

For the quantification of the Cd level, the seedlings were cultivated in growth chambers with a temperature of 20–22 °C as described in the previous section and then the roots of the seedlings were thoroughly washed with distilled water, cut off and dried for three days in 60 °C. Than the samples were sent for analysis using ICP-OES spectrometry to a commercial company (Scallad, Poznań, Poland). The analysis was carried out on samples from three independent biological repetitions.

### Quantification of 8-hydroxyguanosine (8-OHG) in RNA

The 8-OHG quantification in RNA was carried out as previously described^17,18^. Total RNA was isolated using TriReagent (Sigma, USA, T9424) in sterile conditions. Briefly, 1 mL of TriReagent and 2 stainless-steel beads (Qiagen, Germany, 69989) were added to each sample. The samples were homogenized on TissueLyser II (Qiagen, Germany) for 3 min at 25 rounds/min, incubated for 20 min at RT, supplemented with 200 µL of chloroform (Sigma-Aldrich, USA, 32211), thoroughly mixed, incubated for 15 min at RT and centrifuged for 20 min at 4 °C by 12 000 x *g*. The aqueous phase was transferred to new Eppendorf tubes and supplemented with 500 µL of isopropyl alcohol (BioShop, Canada, ISO920.500). Thereafter, the samples were mixed, incubated for 15 min at RT and centrifuged for 15 min at 4 °C by 12 000 x *g*. The aqueous phase was discarded, the pellet washed with 1 mL of 75% ethanol (POCH Basic, Poland, BA6480111) and centrifuged for 5 min at 4 °C by 7 600 x *g*. The ethanol was discarded and the dried pellet dissolved in RNase/DNase free water (BioShop, Canada, WAT333.500). The quality and concentration of the RNA was measured on Synergy LX (BioTek, USA) microplater reader using Take3 Micro Volume Plate.

The level of 8-OHG was quantified using OxiSelect™ Oxidative RNA Damage ELISA-8OHG Quantification Kit (Cell Biolabs, USA, STA-325) according to the manual. Prior to the procedure the samples containing 10 µg of RNA were digested with 20 U of Nuclease S1 (Bio Shop, Canada, NUC333.50) dissolved in 20 mM sodium acetate (Bio Shop, Canada, SAA333.100) for 2 h and with 10 U of alkaline phosphatase from bovine intestinal mucosa (Sigma-Aldrich, USA, P6774-2KU) dissolved in 100 mM TRIS (Sigma-Aldrich, USA, T9424) for further 1 h in 37 °C. In the final step the absorbance of the samples was measured using ˙iMARK™ Microplate Reader (Bio-Rad, USA). The results are expressed as pg of 8-OHG per 1 µg of RNA. The analyses were performed in 3–4 biological repetitions.

### Examination of oxidative stress intensity

The level of glutathione (reduced – GSH and oxidized – GSSG) was quantified using the Glutathione Assay Kit (Cayman Chemicals, USA, 703002) according to the provided protocol. Briefly, approx. 200 mg of roots were homogenized in 1 mL of provided MES buffer for 2 min on Tissue Lyser II (Qiagen, Germany). Thereafter, the samples were centrifuged 15 min at 4 °C by 10 000 x *g*. The supernatant (approx. 800 µL) was supplemented with 10% metaphosphoric acid (MPA, Sigma-Aldrich, USA, 239275), the samples were incubated at 20–22 °C (RT) for 5 min and centrifuged for 5 min at 20–22 °C by 2 000 x *g*. The supernatant was collected to new Eppendorf tubes and 50 µL of 4 M triethanolamine solution (TEAM, Sigma-Aldrich, USA, T58300) was added. The samples were divided into two separate Eppendorf tubes, each containing 600 µL. One tube was used for measurement of GSH and the other tube for measurement of GSSG.

For GSH measurement, 50 µL of samples were transferred to microplate wells and supplemented with 150 µL of Cocktail Mix. In parallel, the standards and blanks containing MES buffer were prepared. The microplate was incubated for 30 min in the dark. Next, the absorbance was measured by Synergy LX (BioTek, USA) microplate reader at 405 nm.

For GSSG measurement, the samples were supplemented with 10 µL of 1 M 2-vinylpyridine (Sigma-Aldrich, USA, 13229-2) and incubated for 60 min at RT. Thereafter, the samples were processed as for GSH measurements – 50 µL of the sample, standard or MES buffer (for the blanks) were transferred to microplate wells, the microplate was incubated for 30 min in the dark at RT and the absorbance measured at 405 nm. The concentration of GSH and GSSG was calculated using Gene 5 software on the basis of standard curve and expressed as for 1 g of FW. The measurements were carried out on three biological replicates.

The level of superoxide anion was measured according to^21^. The roots of seedlings were incubated for 1 h in the dark in a reaction mixture containing 0.05 M potassium buffer, 0.05% NBT (Serva, Germany, 30550.03), 0.1 mM EDTA and 10 mM NaN_3_ (Sigma-Aldrich, USA, 71290). Thereafter, the roots were removed, cut off and weighted. In parallel, the reaction mixture was incubated for 15 min at 85 °C. The absorbance of the solution was measured at 580 nm. The analysis was performed in six biological replicates and the results were expressed as for 1 g of FW.

Lipid peroxidation was estimated on the basis of the changes in the level of thiobarbituric acid reactive substances (TBARS) according to^22^, with minor modifications. Roots of soybean seedlings (approx. 200 mg) were cut off on ice and homogenized with 2 mL of 10% TCA (trichloroacetic acid solution, Sigma, USA, TO699). Samples were centrifuged (10 min, 12 000 x *g* 4 °C), and thereafter 1 mL of the supernatant was transferred to glass tubes. The tubes were supplemented with 4 mL of 0.5% TBA (2-thiobarbutric acid, Sigma, USA, T5500) dissolved in 10% TCA and incubated for 30 min at 95 °C. After cooling on ice, the absorbance of the supernatants was measured at 532 nm and corrected for the nonspecific absorbance at 600 nm. The analysis was performed in three biological replicates.

The content of carbonyl groups reflecting the level of protein carbonylation was measured using the Protein Carbonylation Assay Kit (Simga-Aldrich, USA, MAK094) according to the manual provided by the manufacturer. The absorbance of samples was measured on Synergy LX microplate reader (BioTek, USA) at 375 nm, with guanidine as negative control. For the measurement of protein content, the QuantiPro BCA Assay Kit (Sigma–Aldrich, USA, QPBCA) was used as described in the provided protocol. The measurements were performed in four biological repetitions.

### Detection of 8-OHG enriched transcripts in plant tissues

For immunohistochemical localization of transcripts modified by 8-OHG and reactive oxygen species (ROS), roots (taken from approx. 10 mm behind the tip) were sectioned with the use of vibratome (Leica VT1200S, Germany) while submerged in a buffer bath (PBS, Bioshop, Canada, PBS404). The 100 μm thick sections were fixed in FAA (50% ethanol, 10% formalin, 5% glacial acetic acid), rinsed three times in PBS buffer and then incubated overnight in 4 °C with primary anti-8-OHG antibody (15A3, Santa Cruz Biotechnology, Inc., USA, sc-66036) diluted 1:50 with PBS/1% BSA. Subsequently, the sections were rinsed three times in PBS and then incubated at 37 °C for 2 h with FITC-conjugated anti-mouse secondary antibody (BP-FITC, Santa Cruz Biotechnology, Inc., USA, sc-516140) diluted 1:100 with PBS/1% BSA. For DNA visualization, the material was treated with a solution of SYTO Deep Red (Invitrogen, USA, S34900) at a concentration of 1 µM for 30 min at 37 °C, rinsed with PBS and mounted in antifadent solution AF1 (Citifluor Ltd, UK) on glass slides. Reactive oxygen species were detected with fluorescent dye-based general oxidative stress indicator – 10 µM CM-H2DCFDA (Invitrogen, USA, C6827). Sections were examined under a fluorescence microscope Zeiss Axiostar plus using filterset 09 (Excitation BP 450/490, Emission 515) or an Zeiss LSM510 confocal microscope. On average, five different roots were investigated. The procedures were carried out in the dark by 20–22 °C.

### Preparation of samples for RNA-seq

The poly(A) RNA was purified from the total RNA using GenElute™ mRNA Miniprep Kit (Sigma-Aldrich, USA, MRN 70). Thereafter, 25% of the obtained poly(A)RNA was predestined for sequencing and the remaining 75% for immunoprecipitation (IP). For IP, approx. 7 µg of mRNA were incubated for 2 h in RT with 7 µg of anti-8-OHG antibody (15A3, Sigma-Aldrich, USA, SAB5200010). Thereafter, Protein G Mag Sepharose Beads (20 µL) (Sigma-Aldrich, USA, GE28-9440-08) were added and the samples were incubated for further 15 h in 4 °C. The samples were washed four times with PBS (BioShop, Canada, PBS404) with 0.04% Nonidet™ P40 Substitute (Sigma-Aldrich, USA, 74385) and 8-OHG enriched mRNA was eluted by the addition of 300 µL of PBS with 0.04% Nonident, 30 µL of 10% SDS (BioShop, Canada, SDS005) and 300 µL of phenol-chloroform-isoamyl chloroform (PCI, Sigma-Aldrich, 77617) and incubated for 15 min in 37 °C. The samples were centrifuged (5 min, 15 700 x *g*) and the poly(A)RNA was precipitated by addition of 40 µL of 3 M sodium acetate (BioShop, Canada, SAA333.100), 1 µg of glycogen (Sigma-Aldrich, USA, G1767) and 1 mL of 95% ethanol (POCH Basic, Poland, BA6480111) and incubated in -20 °C overnight. The samples were centrifuged (20 min 14 000 x *g*), washed once with 75% ethanol and dissolved in RNAse/DNase free water (BioShop, Canada, WAT333.500). The negative samples were treated same way with no addition of the antibody.

### RNA sequencing and analysis of sequencing data

The samples included total poly(A)RNA isolated from the roots of control and Cd-treated seedlings (Control and Cd, respectively) and 8-OHG enriched RNA from the control and Cd-treated seedlings (oxyControl and oxyCd, respectively). The samples were derived from three independent biological repetitions and were sequenced by external company (Macrogen, South Korea). The library was prepared using SMART-Seq™ v4 Ultra™ Low Input RNA and the samples were sequenced on NovaSeq 6000 Illumina Platform^23^. The characteristics of the sequenced libraries are presented in Supplementary Table 2. The raw data are deposited in NCBI database under project number PRJNA1158244.

The raw sequences were processed using Trimmomatic^24^ to trim the adapter and low-quality sequences. Then, the soybean genome index was created using STAR^25^ based on the soybean reference genome obtained from Ensemble Plants database (http://plants.ensembl.org/index.html). After mapping the reads to genome using STAR, gene expression was quantified using featureCounts^26^. The low count genes (< 1 CPM) were omitted in further analysis. Differential gene expression analysis was performed in R environment using glimma^27^ and edgeR packages^28^. The list of detected genes was submitted to Zenodo repository: https://zenodo.org/records/13784457. The gene ontology (GO) analysis was carried out using SoyMD platform^29^.

### Statistical analysis

In the case of measurements of RNA oxidation and oxidative stress parameters (the level and ratio of GSH and GSSG, O_2_^·-^, TBARS and protein carbonyl groups), Mann-Whitney U Test was applied to evaluate the statistically significant differences (by *p* = 0.05) between the control and Cd-treated seedlings from specific time point, using Statistica 14.0.0.15 software (TIBCO Software Inc, USA). The non-parametric Mann-Whitney U Test was chosen due to non-normal distribution of the data assessed using Shapiro-Wilk Test^30^.

## Results

As presented in Table 1, Cd was effectively taken up by plants. The level of this metal in the roots of Cd-treated seedlings reached approx. 50–60 µg/g of dry weight (DW). As expected, Cd was not detectable in the roots of the control seedlings.

**Table 1:** The level of Cd (µg/g DW) in the roots of soybean seedlings exposed to Cd at the concentration 10 mg/L (Cd 10) for 1, 2 and 3 h; UD – undetectable. The results are means of 3 biological repetitions ± SE. The results with statistically significant difference in relation to the control are marked with asterisks (*).

	Control	Cd 10
1 h	UD (< 0.051 µg/ g DW)	**61 ± 9** µg/ g DW*
2 h	UD (< 0.051 µg/ g DW)	**65 ± 9** µg/ g DW*
3 h	UD (< 0.051 µg/ g DW)	**49 ± 11** µg/ g DW*

Mean values of 3 repetition± SE are in [bold]

**Fig. 1:**
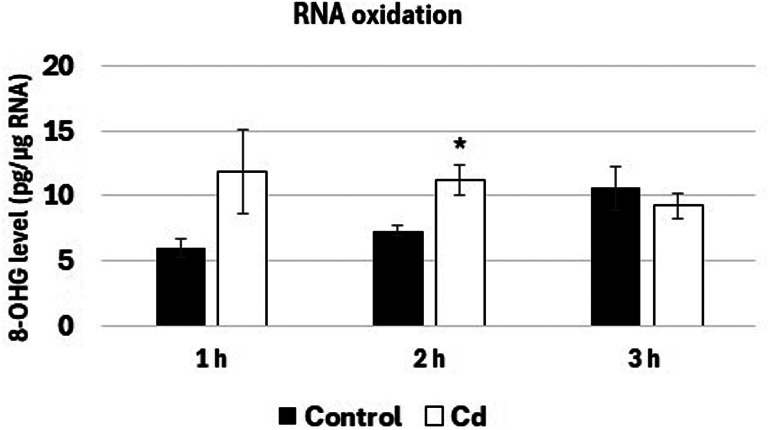
The 8-OHG level in total RNA isolated from the roots of control soybean seedlings (black bars) and roots of seedlings exposed to 10 mg/L Cd (Cd 10, white bars) for 1, 2 and 3 h. The results are means of 3–4 biological repetitions ± SE. The results with statistically significant difference in relation to the control are marked with asterisks (*).

Metal treatment did not affect the morphology nor growth of the seedlings (Supplementary Fig. 1). The short-term exposure to Cd led to slight but statistically significant increase in the level of total RNA oxidation noted after 2 h of treatment (Fig. 1).

**Fig. 2:**
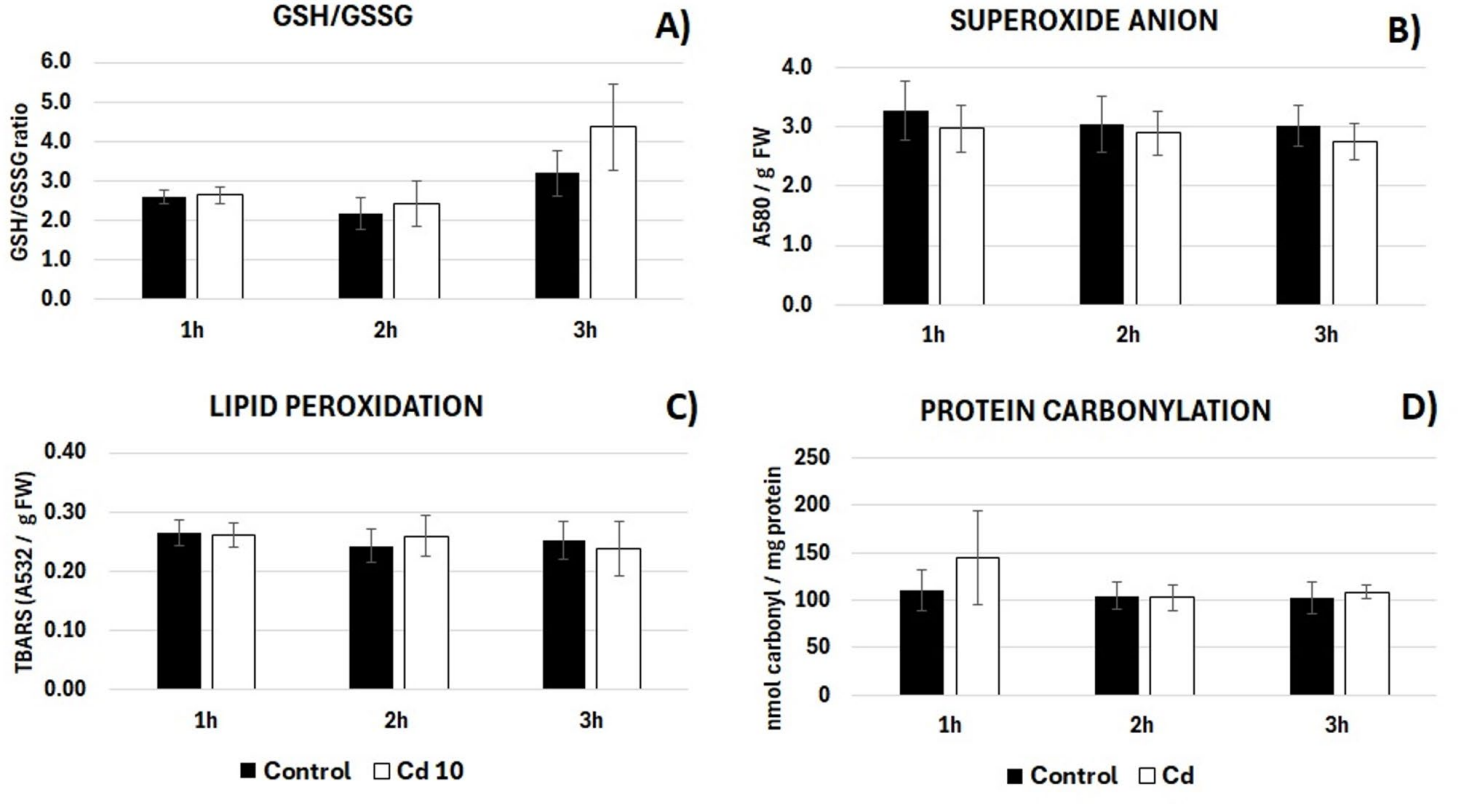
The GSH/GSSG ratio (**A**) and the level of oxidative stress markers - superoxide anion (**B**), TBARS (**C**) and carbonyl groups in proteins (**D**) in the roots of control soybean seedlings (black bars) and roots of seedlings exposed to Cd at the concentration 10 mg/L (Cd 10, white bars) for 1, 2 and 3 h. The results are means of 3–6 biological repetitions ± SE.

On the other hand, exposure to Cd had no effect on the oxidative status of the root cells expressed as the GSH/GSSG ratio (Fig. 2A). The individual levels of GSH and GSSG also did not change in the Cd treatment in a statistically significant way (Supplementary Table 1). Similarly, the short-term Cd treatment had no effect on the level of oxidative stress markers–superoxide anion (Fig. 2B), products of lipid peroxidation (TBARS, Fig. 2C) or carbonyl groups in proteins (Fig. 2D).

**Fig. 3:**
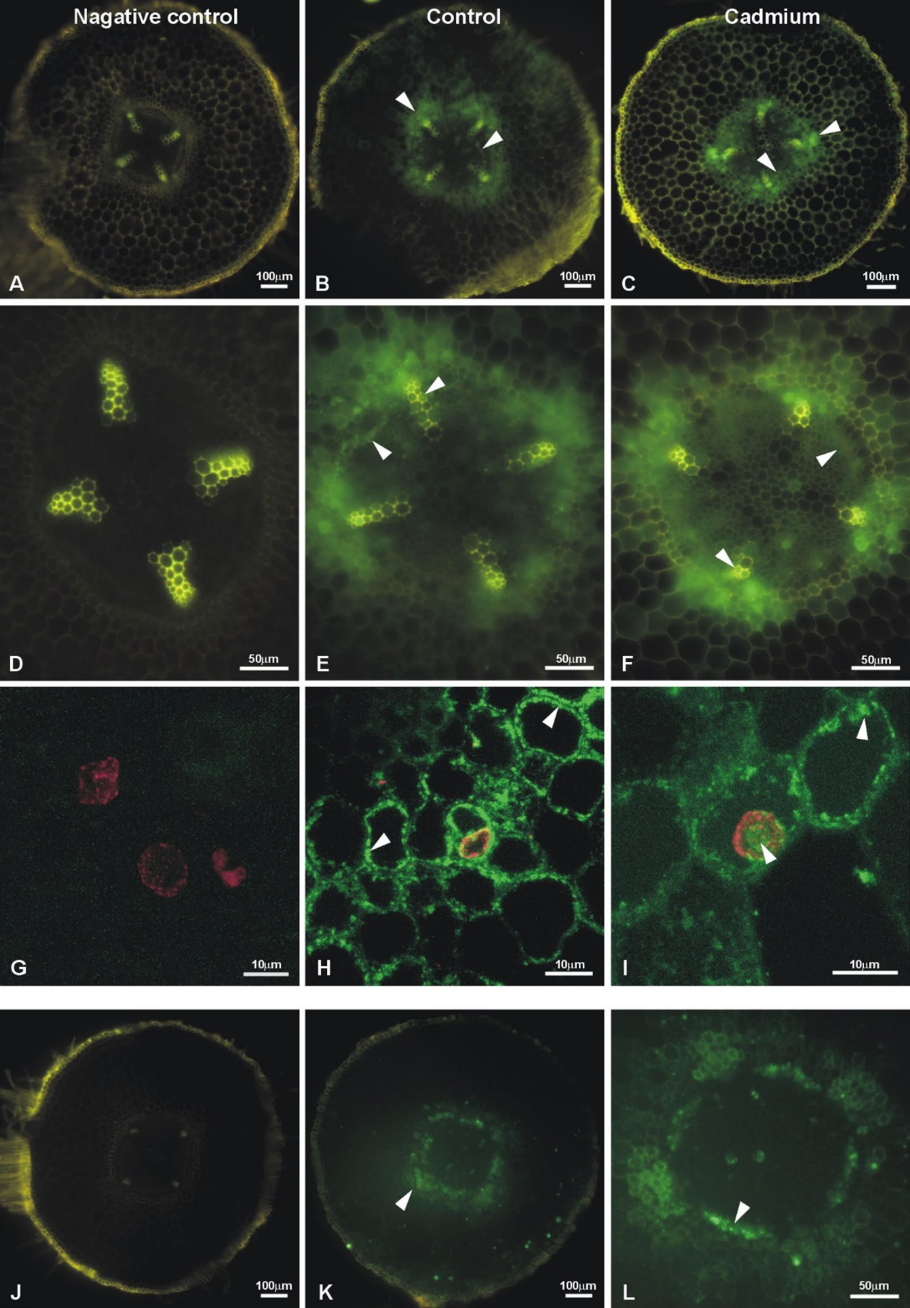
The immunofluorescence signal derived from oxidized nucleic acids (**A**–**I**) and ROS (**J**–**L**) in cross sections of soybean roots. (**A**,** D**,** G**,** J**) – negative control showing autofluorescence in xylem tissue and in epidermis cells; (**B**–**C**,** E**–**F**,** H**–**I**) – accumulation of 8-OHG (white arrowheads) in inner part of cortex, endodermis, xylem and phloem tissue documented on various magnification in control (**B**,** E**,** H**) and in Cd-treated seedlings (**C**,** F**,** I**), the highest magnification shows cells of inner cortex close to endodermis;** (K**–**L**) – ROS detection using 10 µM CM-H2DCFDA in control (**K**) and Cd-treated seedlings (**L**) - distinct signal from some cells of inner part of cortex, endodermis and phloem cells (white arrowheads).

Due to the accumulation of 8-OHG in 2 h of Cd exposure, this time period was selected for the detection of the modification in soybean seedlings’ roots. As shown in Fig. 3, the strongest specific immunoreactive signal for 8-OHG was accumulated in the inner layers of cortex cells and endodermis, especially opposite the primary xylem tissue (Fig. 3B-C). The specific accumulation of 8-OHG was also observed in protoxylem and phloem tissues (Fig. 3E–F). On the single cell level, the specific signal for 8-OHG was accumulated in nucleolus and cytoplasm (Fig. 3H–I). After 2 h of the Cd treatment at concentration of 10 mg/L, there were no distinct differences in signal distribution and accumulation in comparison to cross section of control seedling roots. The fluorescence signal for oxidized transcripts co-localized with ROS detected with 10 µM CM-H_2_DCFDA (Fig. 3K–L).

**Fig. 4:**
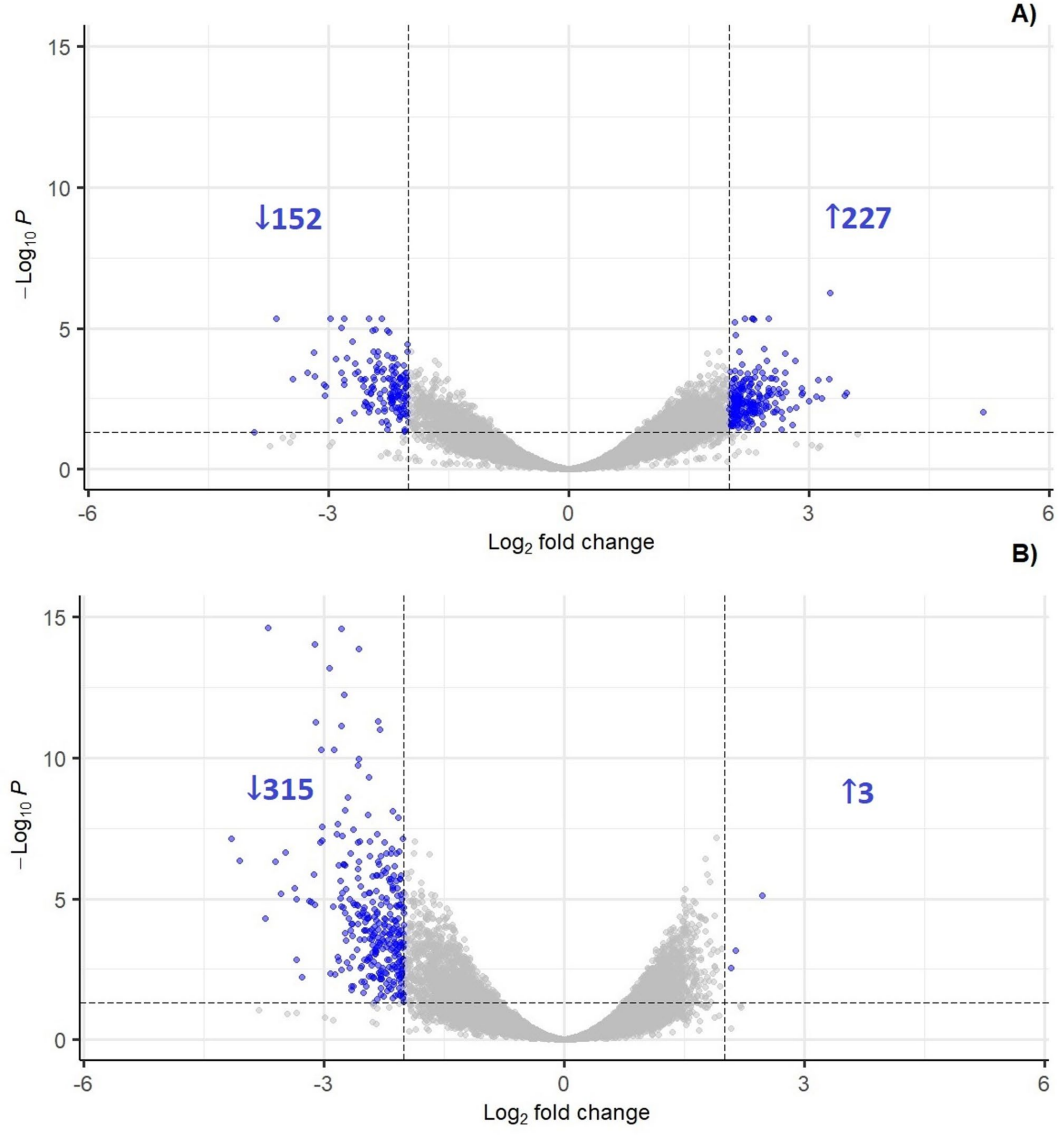
Volcano plot of differentially oxidized transcripts in the roots of control (**A**) and Cd-treated seedlings (**B**), blue dots correspond to transcripts differentially oxidized by adjp < 0.05 showing log_2_ fold change > 2, grey dots correspond to transcripts showing log_2_ fold change < 2 and/or adjp > 0.05.

The results of RNA sequencing show no effect of Cd on the global gene expression by the adjusted *P* < 0.05, neither in samples comprising total poly(A)RNA nor samples containing oxidized poly(A)RNA. On the other hand, there are differences in the intensity of transcripts oxidation in both control and Cd-treated seedlings (Fig. 4). In control, 227 transcripts were identified as highly oxidized (log_2_ fold change > 2; HOTs) and 152 as lowly oxidized (log_2_ fold change ˂− 2; LOTs). Interestingly, in Cd only 3 HOTs were detected, while 315 transcripts were identified as LOTs.

**Fig. 5:**
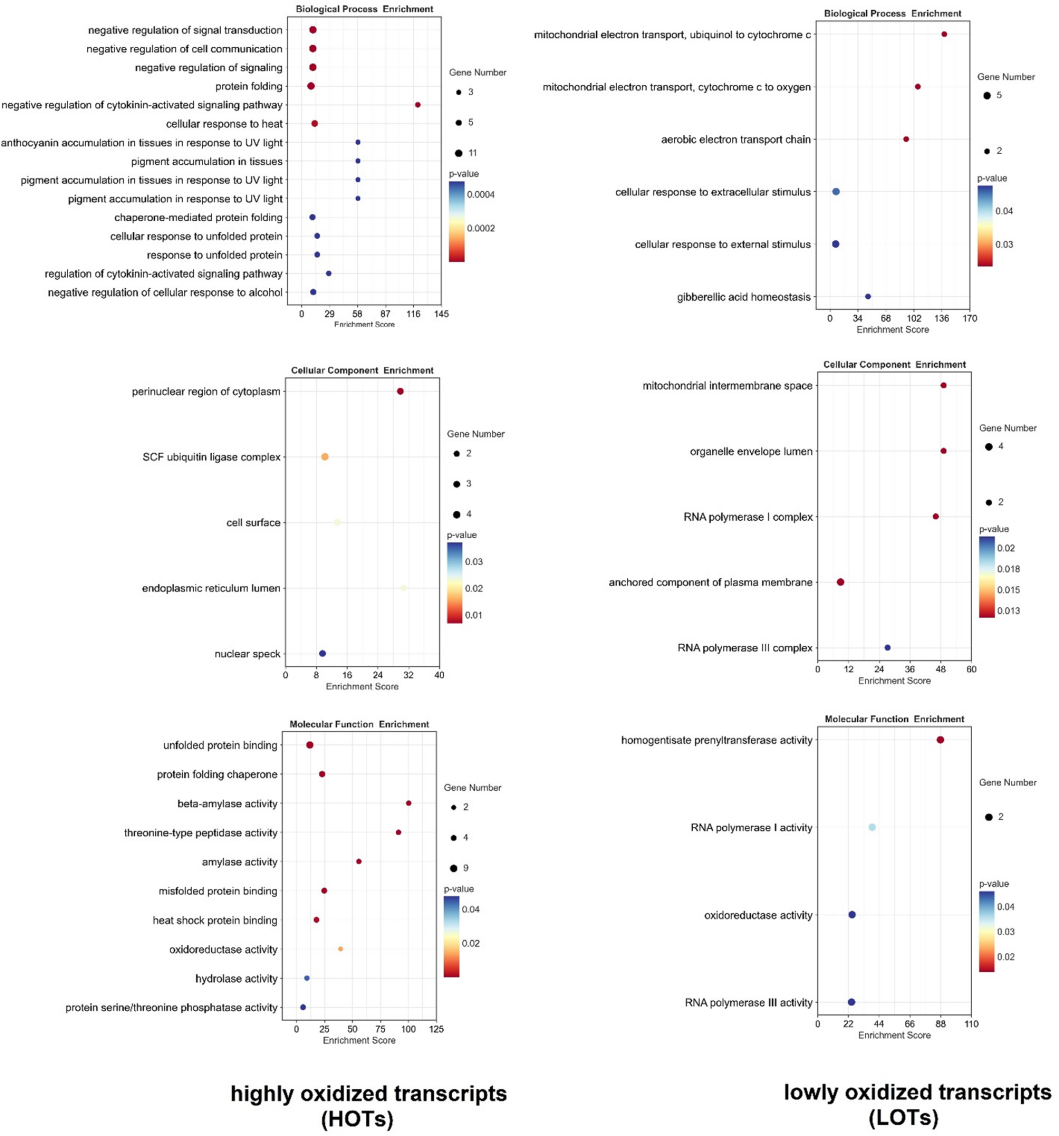
The Gene Ontology (GO) enrichment analysis of the most and least oxidized (log_2_ fold change > 2 and log_2_ fold change ˂− 2, respectively) transcripts in the control samples.

In the control, the HOTs were associated with negative regulation of signalling, stress response and protein folding (Fig. 5). In turn, the LOTs were engaged in the functioning of mitochondria, cellular response to stimuli and homeostasis of gibberellic acid.

**Fig. 6:**
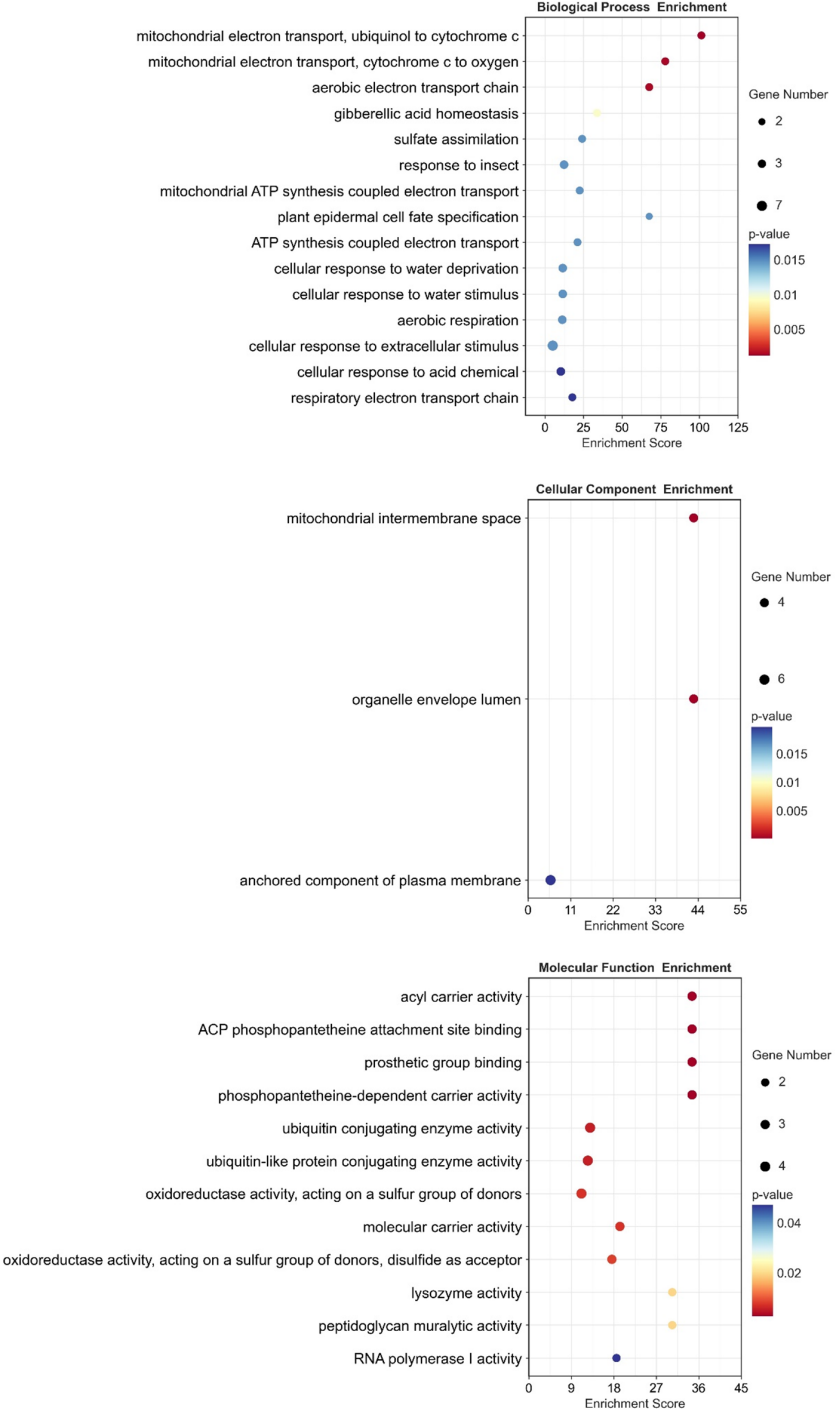
The Gene Ontology (GO) enrichment analysis of the least oxidized transcripts (log fold˂-3), in the Cd-treated samples.

In the Cd treated samples, only three HOTs were found, encoding C2 calcium/lipid-binding and GRAM domain containing protein (GLYMA.17G166900) and two protein kinases (GLYMA.12G097200 and GLYMA.02G257300). Similar to the control, the LOTs in the Cd-treated samples were associated with mitochondria functioning, cellular response to stimuli, gibberellic acid homeostasis, and, in addition, in sulphate assimilation (Fig. 6).

To get better insights into the role of RNA oxidation, individual proteins encoded by the most oxidized transcripts (log_2_ fold change > 3 in control and > 2 in Cd treated samples) were identified (Table 2).

**Table 2:** The list of the most oxidized (log_2_ fold change > 3 in the case of control and > 2 in the case of Cd treatment) transcripts with accession number and encoded protein derived from soybase.

Accession number	Encoded protein	Log_2_ fold change
*Control*		
GLYMA.13G019400	senescence-associated protein	5.2
GLYMA.09G133900	hypothetical protein	3.5
GLYMA.12G155100	unknown protein	3.4
GLYMA.17G166900	C2 calcium/lipid-binding and GRAM domain containing protein; IPR000008 (C2 domain)	3.3
GLYMA.03G144400	seed maturation protein; Late Embryogenesis Abundant protein, LEA-25/LEA-D113	3.3
GLYMA.12G155200	orf129b protein	3.2
GLYMA.11G217200	probable calcium-binding protein CML 18-like	3.2
GLYMA.09G164000	ATP synthase subunit	3.1
*Cd treatment*		
GLYMA.17G166900	C2 calcium/lipid-binding and GRAM domain containing protein	2.5
GLYMA.12G097200	protein kinase superfamily protein	2.1
GLYMA.02G257300	protein kinase family protein	2.1

Similarly, individual proteins encoded by the least oxidized transcripts (log_2_ fold change <− 3) are presented in Table 3.

**Table 3:** The list of the least oxidized (log_2_ fold change < − 3) transcripts with accession number and encoded protein derived from soybase.

Accession number	Encoded protein	Log_2_ fold change
*Control*		
GLYMA.18G00980	B12D protein	− 3.1
GLYMA.08G153600	unknown protein	− 3.2
GLYMA.02G083700	unknown protein	− 3.3
GLYMA.17G117200	early nodulin-related	− 3.5
GLYMA.11G247500	B12D protein	− 3.7
GLYMA.08G362500	pollen protein Ole E I-like protein	− 3.9
*Cd treatment*		
GLYMA.16G153600	arabinogalactan peptide 14-like [*Glycine max*]	− 3.1
GLYMA.02G205100	low temperature and salt responsive protein family	− 3.1
GLYMA.09G245400	cyclin p4; 1	− 3.1
GLYMA.07G258100	uncharacterized protein	− 3.1
GLYMA.08G139800	uncharacterized protein	− 3.2
GLYMA.06G29860	wound-responsive family protein	− 3.2
GLYMA.02G083700	unknown protein	− 3.3
GLYMA.11G060500	uncharacterized protein	− 3.3
GLYMA.04G079200	globin (Oxygen-binding protein) Glb	− 3.3
GLYMA.17G117100	early nodulin-related	− 3.4
GLYMA.12G056700	unknown protein	− 3.5
GLYMA.05G009000	early nodulin-related	− 3.5
GLYMA.20G03610	ribonuclease	− 3.6
GLYMA.18G02200	hypoxia-responsive family protein	− 3.7
GLYMA.08G153600	unknown protein	− 3.7
GLYMA.17G117200	early nodulin-related	− 4.1
GLYMA.11G247500	B12D protein	− 4.2

## Discussion

Despite the important role of ROS in plants functioning and stress response, little is known about the effects of ROS-dependent RNA oxidation in plants. In our previous study, we have shown for the first time that 8-OHG formation in RNA is induced in plants (roots of soybean seedlings) by stress conditions – the Cd treatment^17^. The accumulation was observed after 3 h of treatment and was no longer noted after 24 h. The study aimed to get deeper insights into the metabolism and roles of RNA oxidation in the first three hours of the soybeans’ response to Cd.

An increase in 8-OHG levels was noted after 2 h of Cd exposure (Fig. 1). The difference in the time of 8-OHG accumulation in this (2 h) and previous study (3 h) could depend e.g. on different harvest years of the seeds or the distinct season of the analysis. Nevertheless, both studies confirm early oxidation of RNA in response to Cd. Similarly, rapid RNA 8-OHG accumulation was observed in the roots of soybean seedlings treated with copper (Cu) and lead (Pb)^18^. In addition, elevated RNA oxidation was evidenced by plants response to biotic stresses–nematode infection^20^ and aphids^19^. The cited studies indicate that intensified RNA oxidation might constitute a universal response to abiotic and biotic stress factors.

To get better insights into the general oxidative status of the roots of soybean seedlings, the level of several markers was assessed. The analysis included evaluation of the ratio of reduced to oxidized glutathione (GSH/GSSG), superoxide anion, products of lipid peroxidation and proteins carbonylation (Fig. 2). No differences were noted in the level of the described parameters between the control and Cd-treated seedlings. Similarly, in our previous studies, 3 h exposure to Cd did not induce lipid peroxidation nor general ROS accumulation in soybean seedlings roots^17^. Increased lipid peroxidation and stimulated ROS production was noted only after 24 h of the Cd treatment. In accordance, accumulation of ROS in the prolonged Cd treatment was observed in other plant species including Arabidopsis, pea, rice and lupine^31^. It is suggested that Cd induces several waves of ROS generation^32^. The early wave might be engaged in prime response and signalling events, while ROS accumulation by prolonged metal exposure is associated with oxidative stress and the development of toxicity symptoms. Interestingly, the stress associated ROS waves might be not only timely but also spatially regulated. Various abiotic and biotic stress factors can induce a ROS wave, which propagates through the plant and which is required for the acclimation to unfavourable conditions^33^.

Immunohistochemical detection of 8-OHG revealed that this modification accumulates in the inner layers of cortex cells, endodermis, protoxylem and phloem (Fig. 3B–C, E–F) and co-localizes with ROS accumulation (Fig. 3K–L). The signal was detected in nuclei as well as in cytoplasm (Fig. 3H–I), indicating 8-OHG formation in RNA and DNA. No significant differences were noted between the control and Cd-treated seedlings. The discrepancy between the 8-OHG induction in total RNA in the Cd-treated seedlings measured by the ELISA assays and the lack of differences in the fluorescence signal by the immunohistochemical microscopic detection might result from the differences in the sensitivity of the applied techniques. The observed ROS and consequent 8-OHG accumulation in the endodermis and inner layers of cortex might be associated with developmental processes. Numerous reports show that ROS are regulators of various processes including cell proliferation, transition from proliferation to elongation, expansion due to loosening of cell wall structure, differentiation and programmed cell death^2,34^. In addition, in roots, ROS are involved e.g. in the formation of root hairs, lateral roots, Casparian strips in the endodermis and vascular tissue differentiation^35^. Interestingly, it has been shown in soybean plants and barrel clover (*Medicago truncatula*), that cortex and endodermis are engaged in the development of lateral root primordium^36^. Moreover, also phloem can serve as the source and transport route of ROS. The redox signal can be transmitted *via* phloem as auto-propagation wave with the participation of calcium and nitric oxide^37^. Thus, it is possible that elevated ROS levels observed in the endodermis and/or inner layers of cortex are associated with cell proliferation, initiation of their elongation, formation of Casparian strips or stimulation of lateral roots formation. However, elucidation of the exact role of the observed ROS accumulation in specific tissues of soybean seedlings’ roots needs further experimental investigation. In addition, it is not known if the co-localized accumulation of 8-OHG is just a by-product of ROS action or also a possible regulatory mechanism. To the best of our knowledge, this is the first report showing 8-OHG detection in plant tissues. Earlier, the oxidized RNAs were localized in unicellular green alga *Chlamydomonas reinhardtii*^38^ and in primary rat cortical neuronal cultures^12^. The latter study showed, that the signal was derived mainly from RNA and to a significantly lower extent from DNA^12^.

To identify the oxidized transcripts, the total poly(A)RNA and immunoprecipitated 8-OHG enriched poly(A)RNA were subjected to Illumina sequencing. No global changes in the expression of genes were noted in response to the Cd treatment. Other reports state that, Cd induces significant changes in soybean transcriptome [e.g.^39,40^]. The lack of global modulation of transcriptome in this study, might result from the very short treatment time – seedlings were exposed to Cd for 2 h. In contrast, most of the transcriptomic data are derived from studies applying a prolonged Cd treatment. On the other hand, the results indicate difference in the susceptibility of transcripts to the process of oxidation (Fig. 4). The specific oxidation of certain sets of transcripts was already reported in studies on post-mortem human brain tissues, mice and sunflower and wheat seeds^11–13,15,16^. In addition, it was shown that enrichment in 8-OHG leads to hampered translation^14^ and correlates with a decrease in the level of encoded proteins^16^. In this study, we report increased oxidation of transcripts engaged in negative regulation of signalling, stress response and protein folding (Fig. 5), which might lead to the attenuation of these processes. On the other hand, transcripts involved in mitochondria functioning, response to stimuli and gibberellic acid (GA) homeostasis show significantly lower levels of oxidation (Fig. 6). Their lower susceptibility to oxidation might be associated with increased demand for the encoded proteins during the process of germination and development of young seedlings. In accordance, the least pronounced oxidation (log_2_ fold change < -3) was noted in transcripts encoding proteins involved in the process of nodulation and in the response to hypoxia and low temperature. Again, maintaining stable levels of these proteins might be important for the regulation of germination process and seedlings development. The obtained results indicate that, in present conditions, the specificity of poly(A)RNA oxidation is connected rather with developmental stage than with the Cd treatment. This is most probably dependent on very short metal treatment. Thus, in the future perspective, it would be interesting to identify 8-OHG enriched transcripts in plants exposed to prolonged stress conditions, to elucidate possible changes in the oxidation of stress/defence associated elements. In addition, assessment of the changes in oxytranscriptome in later stages of plants growth could provide more supportive data on possible involvement of RNA oxidation in the regulation of plants development.

Summarizing, this report confirms early stimulation of 8-OHG formation in response to Cd and indicates the tissue and transcript specific formation of this oxidative modification of nucleic acids. However, further studies are still needed, to elucidate the exact role of RNA oxidation in plants development and stress response.

## Electronic supplementary material

Below is the link to the electronic supplementary material.

Supplementary Material 1

## Data Availability

The RNA sequencing raw data are available in NCBI database under the project PRJNA1158244. The list of identified transcripts is available in Zenodo repository https://zenodo.org/records/13784457. Other raw and curated data are available on request from the corresponding authors.
