# Supplementary material for: Oxytranscriptome of soybean seedlings under short-term cadmium treatment
Source: Sci Rep. 2025 Jul 13;15:25308. doi: 10.1038/s41598-025-09324-0 (PMC12256622; doi:10.1038/s41598-025-09324-0)
Supplement: Supplementary file 1 — Supplementary Material 1 [file 41598_2025_9324_MOESM1_ESM.docx]

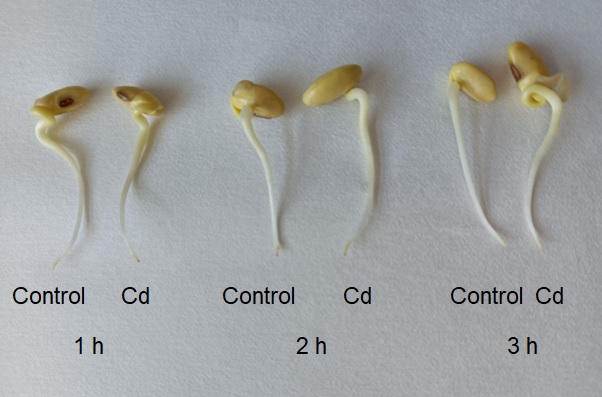


**Supplementary Figure 1**: The morphology of control soybean seedlings and seedlings exposed for 1, 2 and 3h to CdCl_2_ with Cd at the concentration 10 mg/l.

**Supplementary Table 1**: The level of glutathione in reduced (GSH) and oxidized (GSSG) in the roots of control soybean seedlings and seedlings exposed to Cd at the concentration 10 mg/l (Cd10) for 1, 2 and 3 h. The results means of 2-3 biological repetitions ± SE, recalculated for 1 g of FW.

|  | **GSH**  (reduced glutathione) | |
| --- | --- | --- |
|  | **Control** | **Cd10** |
| **1 h** | 71 ± 8 | 61 ± 19 |
| **2 h** | 58 ± 19 | 33 ± 19 |
| **3 h** | 40 ± 12 | 33 ± 15 |
|  | **GSSG**  (oxidized glutathione) | |
|  | **Control** | **Cd10** |
| **1 h** | 27 ± 4 | 23 ± 3 |
| **2 h** | 26 ± 5 | 24 ± 5 |
| **3 h** | 14 ± 5 | 10 ± 6 |

**Supplementary Table 2**: Statistics of sequencing of total poly(A) RNA obtained from the roots of control and Cd-treated (Cd 10) soybean seedlings and of 8-OHG enriched poly(A) RNA isolated from control (oxy Control) and Cd-treated (oxy Cd 10) soybean seedlings.

|  | **Total reads (millions)** | **GC %** | **Q20 %** | **Q30 %** |
| --- | --- | --- | --- | --- |
| **Control** | 113 ± 3.3 | 40.3 ± 1.1 | 93 ± 1.5 | 88 ± 2 |
| **Cd 10** | 107 ± 2.8 | 39.8 ± 1.6 | 93 ± 1.7 | 87 ± 2.2 |
| **oxy Control** | 114 ± 6.2 | 36.9 ± 1.3 | 89 ± 1.1 | 83 ± 1.2 |
| **oxy Cd 10** | 122 ± 1.3 | 41.1 ± 0.4 | 94 ± 0.4 | 88 ± 2.0 |
